# A pre-experimental design evaluation of brief harm reduction interventions to improve coping self-efficacy of carers of people with substance use disorder

**DOI:** 10.1186/s12954-023-00811-z

**Published:** 2023-06-15

**Authors:** Ilze Swanepoel, Gretel Crafford, Stephan Geyer, Tessa S. Marcus

**Affiliations:** 1 Department of Social Work and Criminology, University of Pretoria, Pretoria, South Africa; 2 COPC Research Unit, University of Pretoria, Pretoria, South Africa; 3 Department of Statistics, University of Pretoria, Pretoria, South Africa

**Keywords:** Substance use disorders, Carers, Coping self-efficacy, Brief harm reduction interventions, Problem-focused coping, Emotion-focused coping, Social coping strategies

## Abstract

**Background:**

Globally, the rise in the number of people living with a substance use disorder (SUD) carries a multitude of individual and social health implications for carers and their families, often impacting negatively on their quality of life. Considered from a harm reduction approach, SUD is understood as a chronic protracted, complex health and social condition. From the extant literature, there is no evidence of the harm reduction approach being applied to address the needs of carers/family members who carry the burden of SUD care. This study preliminarily evaluated the Care4Carers Programme. It is a purposively designed set of brief interventions to improve the coping self-efficacy of carers of people with SUD (PwSUD carers) by equipping them to think about ways to exert control over their motivation, behaviours and social environment.

**Methods:**

A pre-experimental, one group pretest–posttest design was implemented with 15 purposively selected participants in the Gauteng Province of South Africa. The intervention was conducted by the lead researcher, a registered social worker. Eight brief intervention sessions were held, over 5–6 weeks at research sites where the participants were identified. The coping self-efficacy scale was completed before and directly after exposure to the programme. Results were analysed using paired *t*-tests.

**Results:**

There were statistically significant (*p* < .05) improvements in carers’ coping self-efficacy, both overall and in respect of each of its constituent components: problem-focused coping, emotion-focused coping and social support strategies.

**Conclusions:**

The Care4Carers Programme improved the coping self-efficacy of carers of people living with SUDs. The application of this programmatic harm reduction intervention to support PwSUD carers should be tested on a larger scale across South Africa.

## Background

Globally, eight to fifteen per cent of people who use substances (approximately 36 million people) live with a substance use disorder (SUD) [1, 2]. In South Africa, the focus of this paper, around 13.3% of people who use substances meet the diagnostic criteria for a SUD [3]. Increased substance use in urban and rural areas has been accompanied by an increase in the demand for treatment services for those who use harmfully across all nine provinces of South Africa [2]. Between 2019 and 2020 the recorded demand for treatment grew nationally by 48.7% from 6317 to 9394, with more than a half of these (54%; 5059) coming from the Gauteng Province [2], the economic hub and most populous of South Africa’s provinces, and the site of this study. Beyond the healthcare needs of people with SUD, SUD has significant psychosocial and economic implications for individuals who care for people living with SUD, as well as other affected family and community members with whom they live. People with SUD require assistance and support from carers as they are unable to routinely care for themselves. In this study, a carer refers to a relative, spouse, or life partner who provides unpaid, informal care for a person with SUD [4]. Although limited, available international and South African research [4, 5] shows that carers of people with SUD face SUD-associated physical, psychological, and emotional health challenges, financial distress and social stress. Recent research has shown that SUD is associated with the weakening of family systems and the deterioration of carer and family quality of life (QOL) [5, 6]. Yet, clinical and social interventions, including those using a harm reduction approach (HR approach) to SUD, rarely address the needs of carers and affected families [7].

Harm reduction is an evidence-based approach to substance use intervention that seeks to reduce the risks of harmful substance use and SUD [8]. It embodies principles of pragmatism, humanistic values, autonomy, and individualism [9–11]. For the carers of people with SUD (PwSUD carers), a HR approach to reduce the negative impacts the disorder has on their physical, mental and social health, needs to involve interventions that focus on their coping strategies. More effective coping strategies could equip them to consider ways to exert control over their motivation, behaviours and social environment [12–14] enabling them to potentially diminish the impact of SUD-associated stressful experiences on their current and future health [15]. Coping strategies are multi-dimensional. Problem-focused coping attempts to reduce or eliminate problems; emotion-focused coping seeks to manage emotional responses to stress [15, 16]; and, social coping strategies refer to people's abilities to elicit societal support [15].

The Care4Carers Programme created through this study, offers an example of a purposively designed brief harm reduction intervention (see Table 1) to capacitate PwSUD carers’ coping self-efficacy (CSE) [15]. CSE refers to an individuals’ belief in their ability to cope with emotions and stressful events. Brief interventions (BI) are an umbrella term for rendering advice and/or counselling [17]. Drawing on heterogeneous theoretical approaches and strategies [18], they aim to change behaviour in the short term. BI involves short (5–60 min), one-on-one encounters, delivered once or over several sessions [19]. BIs in the Care4Carers Programme are guided procedurally by Miller and Rollnick’s (1991) “feedback, responsibility, advice, menu of strategies, empathy, and self-efficacy” (FRAMES) [17, 20]. FRAMES is an approach that combines introspection, learning and empowerment. The BIs were delivered over five to six weeks (with one or two sessions per week) to eligible participants at research sites in the Gauteng Province by the lead researcher who is a registered social worker. The BIs cover problem-focused coping strategies that address harmful substance use, the nature of psychoactive substances and life skills such as conflict management, effective communication, basic financial literacy and problem-solving [15]. Emotion-focused BIs focus on understanding and developing skills that support self-care, mindfulness, stress management and emotional self-regulation. To support social coping strategies, the programme focuses on establishing social support from family, friends and supportive services in the community.

**Table 1:** Outline of Care4Carers programme

#	Session	Aim/goal of the session
1	Education on harmful substance use and psychoactive substances	To promote a basic understanding of what harmful substance use/substance use disorder and psychoactive substances entail
2	Self-care	To enable carers to assess their own self-care needs and to enhance their abilities to practice self-care strategies
3	Coping strategies/skills	To improve carers’ coping mechanisms and ability to adaptively cope in diverse situations
4	Family life and effective communication	To identify the impact of harmful substance use on family functioning and relationships, and to improve family life and relationships and to acquire/improve effective communication skills
5	Mindfulness	To comprehend what mindfulness implies, to identify the impact of harmful substance use on the carers’ spirituality and to enhance carers’ capability to practice mindfulness
6	Basic financial literacy	To assess the financial impact of harmful substance use on the carer, learn how to manage financial strain and acquire effective financial management skills
7	Life skills (stress management, conflict management, emotional regulation)	To improve carers’ capability to cope with stress, to enhance carers’ conflict management strategies, and to enhance carers’ abilities to better regulate their emotions
8	Establishing social support	To understand social support and the benefits thereof, to determine carers' support needs, empower carers to seek support, and provide information on available support services

This paper reports on the results of a pre-experimental design to evaluate the effectiveness of the Care4Carers Programme in improving PwSUD carers’ CSE.

## Methods

The study implemented intervention research to develop and pilot test the Care4Carers Programme [21, 22].

### Design, study setting and sampling

Conducted between June 2021 and March 2022, the pilot study followed a pre-experimental, one group pretest–posttest design [23, 24]. Purposively identified research sites were used to recruit participants, as they provided direct access to PwSUD carers and allowed for indirect recruitment practices. Thus, social workers identified participants using specified inclusion criteria at service sites where PwSUD carers’ family members received treatment for SUD. After sharing information about the content and potential risks and benefits of the programme, they referred carers who expressed interest in the programme to the study. To be included in the research, participants had to be: (1) related to a person with SUD; (2) the primary person providing and caring for the person with SUD; (3) 25 years or older at the time of the study; (4) willing to participate in the study voluntarily; and (5) English literate [23]. PwSUD carers who did not meet one or more of the above criteria were excluded from the research. The study findings are based on the responses of 15 PwSUD carers from the following research sites: three at a public treatment centre in Gauteng (*n* = 3), two at the Department of Social Development, Tshwane office (*n* = 2), and 10 at the Community Oriented Substance Use Programme (COSUP), Tshwane (*n* = 10). The small sample size was dictated by COVID-19-related health protocols and the preliminary nature of the evaluation of the programme (i.e. Phase 4 of intervention research) [21, 22].

### Data collection

Data were collected through the coping self-efficacy scale (CSES). The CSES is a 26-item, 10-point Likert scale which measures people’s confidence or perceived self-efficacy in performing coping behaviours when facing life challenges or threats. It is also used to assess changes in CSE over time [15]. CSE refers specifically to individuals’ beliefs in their ability to cope with stressful events and emotions and is also a means of measuring adaptive or positive coping [25, 26]. It is a standardised, freely available instrument that has been validated for use within the African context [27]. The Likert scale allows participants to express their responses in terms of ordinal-level categories that are ranked along a continuum [23]. The anchors of the 26-item scale are 0 (‘Cannot do at all’), 5 (‘Moderately certain can do’) and 10 (‘Certain can do’). The CSES was self-administered by participants before and directly after exposure to the programme.

### Data analysis

Data were analysed in consultation with a statistician using SAS 9.4. Descriptive statistics, including the mean score was calculated for each construct for both pre-and post-test measurements. The mean score may be regarded as a representative measure and was investigated further during statistical analyses. Apart from the overall CSE, the three constructs measured through the CSES are participants’ confidence in their abilities to use problem-focused, emotion-focused and social coping strategies [15]. Paired t-tests were performed to evaluate whether significant differences could be determined between the post- and pre-test measurements for the overall CSE and the three constructs. An investigation of normality in the differences in the mean scores between the pre- and post-test scores revealed no significant deviations from normality and supported the use of this test [28, 29]. The CSE and the three constructs provided Kolmogorov–Smirnov tests for normality with p-values greater than 0.05 [30].

The reliability of the CSES was confirmed by calculating Cronbach’s alpha coefficient. The values were as follows; *α* = 0.87 at pretest and *α* = 0.92 at posttest for overall CSE, *α* = 0.77 and *α* = 0.82 for problem-focused coping, *α* = 0.80 and *α* = 0.91 emotion-focused coping, and *α* = 0.27 and *α* = 0.75 for social coping. Holistically seen, the CSES is therefore considered to be reliable within the context of the present study [31, 32].

## Results

The 15 participants had an average age of 45 years (range: 28–65 years; *SD* = 10.5) and all identified as female.

The descriptive statistics pertaining to the pre- and post-test measurements for the CSES are presented in Table 2.

**Table 2:** Descriptive statistics for the pre-and post-test measurements of the CSES (*n* = 15)

Coping construct measured	Minimum	Maximum	Mean (*M*)	*SD*
*Overall CSE*				
Pre-test	4.58	7.50	5.51	0.81
Post-test	6.23	9.35	7.62	0.88
*Problem-focused coping*				
Pre-test	4.17	7.42	5.43	0.83
Post-test	5.92	9.25	7.69	0.84
*Emotion-focused coping*				
Pre-test	3.89	7.89	5.47	1.03
Post-test	5.22	9.44	7.73	1.18
*Social coping strategies*				
Pre-test	4.40	7.00	5.75	0.95
Post-test	4.40	10.00	7.27	1.29

Statistically significant differences found in this study show that the brief interventions improved overall CSE, as well as in respect of each of the individual components of coping that were measured (see Table 3).

**Table 3:** Paired t-test results

Coping construct	Paired differences				
	*M*	*SD*	*t* statistic	d*f*	*p*-value (One-sided)
*Overall coping self-efficacy*					
Post–pretest	2.12	1.27	6.48	14	< .0001*
*Problem-focused coping*					
Post-pretest	2.26	1.17	7.47	14	< .0001*
*Emotion-focused coping*					
Post–pretest	2.27	1.57	5.61	14	< .0001*
*Social coping strategies*					
Post–pretest	1.52	1.53	3.86	14	0.0017*

*p < .05

The results of the paired t-test compared the PwSUD carers’ coping self-efficacy before and after exposure to the programme. The average difference between the post- and pre-test measurements for the overall CSE was (*M* = 2.12, *SD* = 1.27). This improvement was statistically significant, *t*(14) = 6.48, *p* < 0.0001. The average difference between the post- and pre-test measurements for each of the constructs measured by the CSES was statistically significant (*M* = 2.26, *SD* = 1.17), *t*(14) = 7.47, *p* < 0.0001 for problem-focused coping; (*M* = 2.27, *SD* = 1.57), *t*(14) = 5.61, *p* < 0.0001 for emotion-focused coping; and, *t*(14) = 3.86, *p* = 0.0017 for social coping strategies (*M* = 1.52, *SD* = 1.53).

## Discussion

The study found that the Care4Carers Programme improved the overall and component-specific CSE of PwSUD carers. Improved CSE results indicate that participants perceived themselves as being better able to cope with SUD-related problems, both as a result of changes in their understanding of the chronic nature of SUD and how substances work, and because the programme provided them with self-care and other valuable life skills. Their emotion-focused coping improved the most, a component that previous research has shown to be the most responsive to change when people have little control over situations [33]. Supportive interventions, including accepting the disorder through knowledge acquisition, practising self-care, mindfulness, effective coping strategies, and emotional regulation skills assisted them in better regulating their feelings and helping restore their psychological well-being [33, 34]. There was less improvement in participants’ social coping, partly because PwSUD carers feel less in control of the social support environment, especially given widespread prejudice and stigma towards substance use and mental health [35].

PwSUD carers’ CSE is particularly important as people’s belief in the extent of their ability to cope is predictive of their levels of coping behaviour as well as being a prerequisite to changing coping behaviour [15]. Adaptive coping occurs when people achieve a better ‘fit’ between stressful events and their coping strategies which in turn, is predictive over time of a reduction in psychological distress and increased health and psychological well-being [36]. People’s belief in their coping abilities is crucial to implementing adaptive coping strategies [27]. The CSES scores are attributed to changes in an individual’s confidence in their general ability to cope, without reference to specific stressful events [15]. By helping participants think about how to deal with their challenges, the Care4Carers Programme improved PwSUD carers’ confidence in and ability to use strategies and skills with a better ‘fit’ to their needs. Higher CSE is associated with a multitude of positive health outcomes, including decreased depression, anxiety, psychological stress, burnout [26, 37–39] and improved physical health [40], resulting in decreased burden [33].

Studied across diverse research fields, Bandura [41] has described perceived self-efficacy “as a form of perceived operative capability”, i.e. a person’s belief about what they can do with the resources they have in the circumstances they find themselves in. For PwSUD carers who continuously face new or unresolved stressful situations, improving their operative capability, by developing their reflective, informational and practical skills could help them make choices that positively contribute to improved well-being [27, 41].

The findings of this study are limited by the small number of participants, the absence of male carers in the intervention and the use of English in a multi-lingual society. There, therefore, is a need to determine the promise of the intervention by evaluating its effectiveness (phase 5 and 6 of intervention research) using representative samples across South Africa.

## Conclusions

In the field of SUD, this study provides novel insights into the positive role of programmatic harm reduction interventions that focus on the CSE of PwSUD carers. The findings show that the Care4Carers Programme improved the CSE of carers of PwSUD by developing their confidence in and abilities to apply better fit and adaptive coping behaviours. Improved CSE, in turn, can be expected to contribute to better biopsychosocial health outcomes, a reduction in the burden of caregiving, and enhanced PwSUD carer QOL. This is particularly important given the chronic nature of SUD, the rise in PwSUD numbers, across the country and the fact the burden of care is borne by mostly female family members.

## Data Availability

The dataset generated and analysed during the current study is available in the Figshare repository, https://doi.org/10.25403/UPresearchdata.21904644.v1.

## Abbreviations

BI: Brief interventions
HR approach: Harm reduction approach
SUD: Substance use disorder
PwSUD carers: People with substance use disorder carers
UNODC: United Nations Office on Drugs and Crime
SACENDU: South African Community Epidemiology Network on Drug Use
CSE: Coping self-efficacy
CSES: Coping self-efficacy scale
COSUP: Community Oriented Substance Use Programme
QOL: Quality of life

## References

[1] United Nations Office on Drug and Crime. World Drug Report 2021 [Internet]. Austria: Division for Policy Analysis and Public Affairs United Nations Office on Drugs and Crime; 2021 June [cited 2022 May 01]. Available from: https://www.unodc.org/res/wdr2021/field/WDR21_Booklet_2.pdf.

[2] South African Community Epidemiology Network on Drug Use (SACENDU). Monitoring alcohol and drug abuse treatment admissions in South Africa: July to December 2020 (Phase 49) [Internet]. Tygerberg: SACENDU; 2021 Dec [cited 2022 May 01]. Available from: https://www.samrc.ac.za/sites/default/files/attachments/2022-0215/SACENDU%20Phase%2049%20full.pdf.

[3] Myers B, Kock JR, Johnson K, Harker N (2022). Factors associated with patient-reported experiences and outcomes of substance use disorder treatment in Cape Town. S Afr Addict Sci Clin Pract.

[4] Jackson S. Coping Methods: Primary carers of adults who are dependent on illegal substances. [unpublished dissertation]. Bellville: University of Western Cape; 2012.

[5] Tabeleão VP, Tomasi E, Quevedo L (2014). Burden on relatives of people with psychic disorder: levels and associated factors. Revista De Psiquiatria Clinica.

[6] Marcon SR, Rubira EA, Espinosa MM, Belasco A, Barbosa DA (2012). Quality of life and stress in caregivers of drug-addicted people. Acta Paulista de Enfermagem.

[7] Scheibe A, Sibeko G, Shelly S, Rossouw T, Zishiri V, Venter W (2020). Southern African HIV Clinicians Society guidelines for harm reduction. South Afr J HIV Med.

[8] Van Wormer K, Davis DR (2018). Addiction treatment: a strengths perspective.

[9] Hawk M, Coulter RWS, Egan JE, Fisk S, Friedman MR, Tula M, Kinsky S (2017). Harm reduction principles for healthcare settings. Harm Reduct J.

[10] Gaetz S. A pragmatic, humanistic and effective approach to addictions: The importance of harm reduction. In The Inclusion Working Groups, Canadian Observatory on Homelessness, editors. Homelessness is only one piece of the puzzle: Implications for Policy and Practice. Canada: Canadian Observatory on Homelessness Press; 2015. pp. 104–112.

[11] Gray A (2017). Harm reduction: a neglected policy option in South Africa. SA Pharm J.

[12] Bandura A (1997). Self-efficacy: toward a unifying theory of behavioral change. Psychol Rev.

[13] Bandura A (1986). The explanatory and predictive scope of self-efficacy theory. J Soc Clin Psychol.

[14] Bandura A (1997). Self-efficacy: the exercise of control.

[15] Chesney MA, Neilands TB, Chambers DB, Taylor JM, Folkman S (2006). A validity and reliability study of the coping self-efficacy scale. Br J Health Psychol.

[16] Baqutayan SMS (2015). Stress and coping mechanisms: a historical overview. Mediterr J Soc Sci.

[17] Platt L, Melendez-Torres GJ, O'Donnell A, Bradley J, Newbury-Birch D, Kaner E, Ashton C (2016). How effective are brief interventions in reducing alcohol consumption: do the setting, practitioner group and content matter? Findings from a systematic review and metaregression analysis. BMJ Open.

[18] Rosembaun A, Rojas P, Rodriguez MV, Barticevic N (2018). Brief interventions to promote behavioral change in primary care settings, a review of their effectiveness for smoking, alcohol and physical inactivity. Medwave.

[19] Mattoo SK, Prasad S, Ghosh A (2018). Brief interventions in substance use disorders. Indian J Psychiatry.

[20] Rodgers C (2018). Brief interventions for alcohol and other drug use. Aust Prescr.

[21] Thomas EJ, Rothman J, Rothman J, Thomas EJ (1994). An integrative perspective on intervention research. Intervention research: design and development for human service.

[22] Fraser MW, Galinsky MJ (2010). Steps in intervention research: designing and developing social programs. Res Soc Work Pract.

[23] Neuman WL (2014). Social research methods: qualitative and quantitative approaches.

[24] Fouché CB, Roestenburg WJH, Fouché CB, Strydom H, Roestenburg WJH (2021). Quantitative research designs. Research at Grass Roots for the social sciences and human services professions.

[25] Midkiff MF, Lindsey CR, Meadows EA (2018). The role of coping self-efficacy in emotion regulation and frequency of NSSI in young adult college students. Cogent Psychol.

[26] Rodkjaer L, Chesney MA, Lomberg K, Ostergaard L, Laursen T, Sodemann M (2013). HIV-infected individuals with high coping self-efficacy are less likely to report depressive symptoms: a cross-sectional study from Denmark. Int J Infect Dis.

[27] Van Wyk MM. Validation of a coping self-efficacy scale in an African context. [unpublished dissertation]. Potchefstroom: North-West University; 2010.

[28] Bartley A, Hashemi L, Fouché CB, Strydom H, Roestenburg WJH (2021). Quantitative data analysis and interpretation. Research at Grass Roots for the social sciences and human services professions.

[29] Pietersen J, Maree K, Maree K (2016). Overview of some of the most popular statistical techniques. First steps in research.

[30] Privitera GJ (2016). Essential statistics for the behavioural sciences.

[31] Heale R, Twycross A (2015). Validity and reliability in quantitative studies. Evid Based Nurs.

[32] Pietersen J, Maree K, Maree K (2016). Standardisation of a questionnaire. First steps in research.

[33] Hoel TL, Geirdal AØ (2016). Burden, coping and mental health among the next of kin of people with a substance abuse problem. Sykepleien Forskning.

[34] Ryff CD (2014). Psychological well-being revisited: advances in the science and practice of eudaimonia. Psychother Psychosom.

[35] Committee on the Science of Changing Behavioral Health Social Norms, Board on Behavioral, Cognitive, and Sensory Sciences, Division of Behavioral and Social Sciences and Education, National Academies of Sciences, Engineering, and Medicine. Ending Discrimination Against People with Mental and substance use disorders: The Evidence for Stigma Change. Washington, D.C.: National Academies Press; 2016. 27631043

[36] Tran T, La N, Nguyen H, Shochet I, Nguyen N, Wurfl A, Orr J, Ngunyen H, Stocker R, Fisher J (2022). Validation of the coping self-efficacy scale: Vietnamese version for adolescents. BMC Psychol.

[37] Melato SR, Van Eeden C, Rothmann S, Bothma E (2017). Coping self-efficacy and psychosocial well-being of marginalised South African youth. J Psychol Afr.

[38] Hau JN. Coping self-efficacy influences health information avoidance. [unpublished dissertation]. Merced: University of California; 2019.

[39] Wahlberg L, Nirenberg A, Capezuti E (2016). Distress and coping self-efficacy in inpatient oncology nurses. Oncol Nurs Forum.

[40] Denton FN, Rostosky SS, Danner F (2014). Stigma-related stressors, coping self-efficacy, and physical health in lesbian, gay, and bisexual individuals. J Couns Psychol.

[41] Bandura A (2007). Much ado over a faulty conception of perceived self-efficacy grounded in faulty experimentation. J Soc Clin Psychol.

